# The sustainability of new programs and innovations: a review of the empirical literature and recommendations for future research

**DOI:** 10.1186/1748-5908-7-17

**Published:** 2012-03-14

**Authors:** Shannon Wiltsey Stirman, John Kimberly, Natasha Cook, Amber Calloway, Frank Castro, Martin Charns

**Affiliations:** 1 Women's Health Sciences Division, National Center for PTSD, Boston, MA, USA; 2 VA Boston Healthcare System, Boston, MA, USA; 3 Department of Psychiatry, Boston University, Boston, MA, USA; 4 Department of Healthcare Management, The Wharton School of the University of Pennsylvania, Philadelphia, PA, USA; 5 VA Center for Organization, Leadership, and Management Research, Boston, MA, USA; 6 Department of Health Policy and Management, Boston University School of Public Health, Boston, MA, USA

## Abstract

**Background:**

The introduction of evidence-based programs and practices into healthcare settings has been the subject of an increasing amount of research in recent years. While a number of studies have examined initial implementation efforts, less research has been conducted to determine what happens beyond that point. There is increasing recognition that the extent to which new programs are sustained is influenced by many different factors and that more needs to be known about just what these factors are and how they interact. To understand the current state of the research literature on sustainability, our team took stock of what is currently known in this area and identified areas in which further research would be particularly helpful. This paper reviews the methods that have been used, the types of outcomes that have been measured and reported, findings from studies that reported long-term implementation outcomes, and factors that have been identified as potential influences on the sustained use of new practices, programs, or interventions. We conclude with recommendations and considerations for future research.

**Methods:**

Two coders identified 125 studies on sustainability that met eligibility criteria. An initial coding scheme was developed based on constructs identified in previous literature on implementation. Additional codes were generated deductively. Related constructs among factors were identified by consensus and collapsed under the general categories. Studies that described the extent to which programs or innovations were sustained were also categorized and summarized.

**Results:**

Although "sustainability" was the term most commonly used in the literature to refer to what happened after initial implementation, not all the studies that were reviewed actually presented working definitions of the term. Most study designs were retrospective and naturalistic. Approximately half of the studies relied on self-reports to assess sustainability or elements that influence sustainability. Approximately half employed quantitative methodologies, and the remainder employed qualitative or mixed methodologies. Few studies that investigated sustainability outcomes employed rigorous methods of evaluation (e.g., objective evaluation, judgement of implementation quality or fidelity). Among those that did, a small number reported full sustainment or high fidelity. Very little research has examined the extent, nature, or impact of adaptations to the interventions or programs once implemented. Influences on sustainability included organizational context, capacity, processes, and factors related to the new program or practice themselves.

**Conclusions:**

Clearer definitions and research that is guided by the conceptual literature on sustainability are critical to the development of the research in the area. Further efforts to characterize the phenomenon and the factors that influence it will enhance the quality of future research. Careful consideration must also be given to interactions among influences at multiple levels, as well as issues such as fidelity, modification, and changes in implementation over time. While prospective and experimental designs are needed, there is also an important role for qualitative research in efforts to understand the phenomenon, refine hypotheses, and develop strategies to promote sustainment.

## Background

All systems and organizations are faced with the challenge of implementing new practices at one time or another, yet many of the innovations that are initially successful fail to become part of the habits and routines of the host organizations and communities. Why do some take root and flourish while others languish? Recognizing the need to promote the use of best practices to achieve better outcomes in healthcare, many government agencies and community organizations have devoted significant resources to promoting research on evidence-based practices (EBPs), clinical guideline implementation, and quality-improvement programs [1,2]. The National Institutes of Health, for example, have given priority to research on the implementation of best practices and evidence-based interventions, and many systems and communities have endeavored to implement specific healthcare interventions or programs to promote improved health outcomes. One consequence of these emerging priorities is the rapid development of the field of implementation science. Most studies in this field thus far have focused on identifying the factors that are critical to the success of initial implementation efforts. While this is a promising start, policy makers and other stakeholders are increasingly concerned with the long-term impact of their investment. However, as Greenhalgh and her colleagues (2004) pointed out in their review of the dissemination and implementation literature, there is a "near absence of studies focusing primarily on the sustainability of complex service innovations" [3].

The results of program evaluation and research to date suggest that sustainability must be studied as a distinct and dynamic phenomenon [4,5]. Although a variety of factors may create conditions that facilitate initial implementation, their presence or influence may diminish over time [6-8]. Even when initial implementation efforts are successful, interventions or programs do not necessarily continue as originally implemented. At times, discontinuation of a particular intervention may be the result of development or discovery of more effective, efficient, or compatible practices [9]. Adaptations, partial continuation of a program or intervention, or integration of new practices may occur in response to new evidence, changes in priorities or resource availability, or other contextual influences. At other times, however, failure to maintain an effective program or intervention at a sufficient level of quality, intensity, or comprehensiveness once implemented is at odds with the original goals and intentions of the host systems or organizations [10-12]. New practices may simply be added on top of existing ones rather than becoming fully integrated [13], which may make them particularly vulnerable to erosion over time [14]. Unintentional "slippage" can occur as a result of factors such as local staffing conditions, lack of resources, or competing demands [4]. If these processes result in failure to achieve desired outcomes, negative appraisals of the value of the interventions themselves [15] can in turn make discontinuation more likely. Understanding these processes and determining how to foster the continuation of effective practices at a level that is sufficient to yield desired health outcomes is at least as important as understanding how to implement them in the first place [16].

Many factors make it difficult to study sustainability and draw conclusions in the current literature. A fundamental challenge is the tension that exists between the continuation of interventions as originally designed and the need to adapt them for use in contexts that may differ in important ways from those in which they were originally developed and tested [5,16,17]. A number of conceptualizations of sustainability have been proposed that reflect differing priorities and perspectives on this issue [18]. In some models, the intervention, rather than the system into which it is introduced, is the focal point of interest. Such models tend to identify a set of factors or conditions that increase the likelihood of sustainability of a specific intervention [17]. This approach is very different from models and studies that examine sustainability from an ecological or complex-systems perspective. These models emphasize the interconnection between broader environmental forces, contextual influences, and the program or intervention itself [19,20]. The differing approaches have important implications for the way that research is conducted and the conclusions that can be drawn. For example, the former perspective may reflect an emphasis on determinants of the preservation, fidelity to, or discontinuation of a program or intervention. In contrast, research conducted from an ecological perspective would seek to understand the ways in which the intervention and the local context mutually adapt and evolve [21] and how this process impacts sustainability. Additional challenges to the study of sustainability and interpretation of the literature include the numerous definitions and related but not entirely equivalent terms that have been used in differing fields, and variation in the timing and method of assessment employed across studies. Furthermore, the assessment of programs, practices, and interventions as varied as community-level prevention programs, medical records systems, psychotherapies, and quality-improvement programs will necessarily limit the extent to which assessment can be standardized.

To better understand the state of research on sustainability to date, we reviewed studies that investigated whether or to what extent programs or interventions that had previously been implemented were sustained, and those that sought to understand factors that influence their sustainment. We present an overview of the ways that some key research considerations have been addressed from this perspective in a variety of fields, and we allow what we found using this approach to guide our synthesis of the results and recommendations. For the purposes of this review, we consider relevant studies to be those that identified interventions, procedures, or programs that were implemented to achieve specific program-, patient-, or population-level benefits. We reviewed studies that examined (1) sustainability outcomes [22] (such as the continuation of some or all components [23] or the desired recipient-level outcomes that occurred after initial efforts to implement, fund, or study a new practice were complete) or (2) influences on the sustainment of these programs or innovations. This review included studies that used a variety of terms to describe sustainability (e.g., "maintenance," "durability," "institutionalization," and "routinization" [9,24,25]) and the decision to discontinue or the failure to sustain programs or interventions (e.g., "de-adoption," "divestment," "exnovation," and "discontinuation" [6,26,27]). However, for the purpose of consistency in the current review, we will primarily use the terms "sustainability" (or "sustainment") and "discontinuation," respectively. Specific questions that guided our review include the following:

• How has sustainability been defined?

• At what levels and units of analysis has it been studied?

• What research methods have been used?

• Over what time periods?

• What outcomes have been reported in the empirical literature?

• What were the findings?

• What has research told us to date about influences on sustainment?

Our findings provide an overview of the current state of the research literature on the sustainment of specific interventions and programs that were implemented to achieve particular goals or benefits. By looking broadly at efforts to study the phenomenon, it may be possible to distill those considerations that should be integral to programs of research that examine the sustainability of specific interventions, programs, and practices [23]. Based on these findings, we will make a number of recommendations for defining, assessing, and studying this topic in future research.

## Methods

### Search method

We searched the MEDLINE, ISI, PsycINFO, Academic Search Premier, Health Source, ERIC, and Google Scholar databases using the terms "sustainability," "implementation," "long-term implementation," "routinization," "discontinuation," "de-adoption," "durability," "institutionalization," "maintenance," "capacity building," and "knowledge utilization." Truncated forms of these terms (e.g., "sustai*", "routini*", "institutionali*") and alternative spellings were included in the search. We also employed a snowballing strategy, in which we searched the reference sections of reviews and theoretical papers on implementation and sustainability [2-4,19,23,25,28,29] and those found in our review. We searched the tables of contents of key journals and journals that had published more than one relevant study on sustainability. These journals included the following: *Academy of Management Review, Academy of Management Journal, Administrative Science Quarterly, American Journal of Public Health, Administration and Policy in Mental Health and Mental Health Services Research, American Journal of Evaluation, Implementation Science, Health Services Management Research, Health Services Research, Healthcare Management Review, Journal of Healthcare Management, The Journal of Nursing Administration, The Journal of General Internal Medicine, Medical Care Research and Review, Millbank Quarterly*, and *Psychiatric Services*. Additionally, we examined papers that had cited influential models or reviews of implementation or sustainability [2,3,5,19,25,30-32]. Finally, we provided the list of articles that were found using these strategies to four individuals known to the investigators who study implementation or sustainability and asked them to share additional articles that they were aware of that had not been included. This yielded nine additional studies.

### Inclusion and exclusion criteria

Our inclusion criteria included peer-reviewed studies that addressed sustainability of specific interventions or programs, were written in English, and were published or in press by July 2011. Because sustainability has been defined in numerous ways, we included all studies in which the authors used one of the terms described above or in which an effort was made to determine the extent to which a program or intervention continued after an initial period of training, implementation, or study. Studies were coded only if they included a methodology or procedure designed to identify (1) the status of the program after the initial implementation effort or funding has ended (e.g., fidelity, percent implemented, presence or absence of key components, or discontinuation); (2) the program-, service-, or recipient-level outcomes measured after external support or funding was withdrawn; or (3) the influences on the persistence of the implementation, whether or not the primary focus of the article was sustainability. Articles were excluded if they (1) reported only on initial implementation efforts, (2) were purely narrative accounts or papers on "lessons learned" that did not examine sustainability using qualitative or quantitative research methodologies, (3) reported only long-term follow-up of individuals after a clinical trial or intervention study, or (4) contained insufficient information to determine whether inclusion or exclusion criteria were met (e.g., ambiguity or failure to report the timeframe during which measures were collected). Studies were considered to focus on initial implementation efforts if the original training, supervision, monitoring, or funding support was ongoing throughout the time period of the research (unless monitoring was considered a central element of the program or conducted strictly to assess sustainability, with minimal or no feedback provided).

### Review methods

All titles and abstracts retrieved by electronic searching were reviewed by one reviewer, who screened out papers that were not related to implementation (e.g., articles related to sustainable agriculture or discontinuation of medications in the context of clinical interventions). Where it was not possible to exclude articles based on title and abstract, full text versions were obtained and their eligibility was assessed. Full text versions of all potentially relevant articles identified from the reference lists of included articles were obtained. Papers related to implementation were screened independently by two reviewers, and those studies that did not meet the inclusion criteria were excluded. Raters agreed on 95% of the papers that were excluded and agreed on the reasons for exclusion for 91% of the articles. Disagreements were resolved through discussion and consensus.

**Figure 1:**
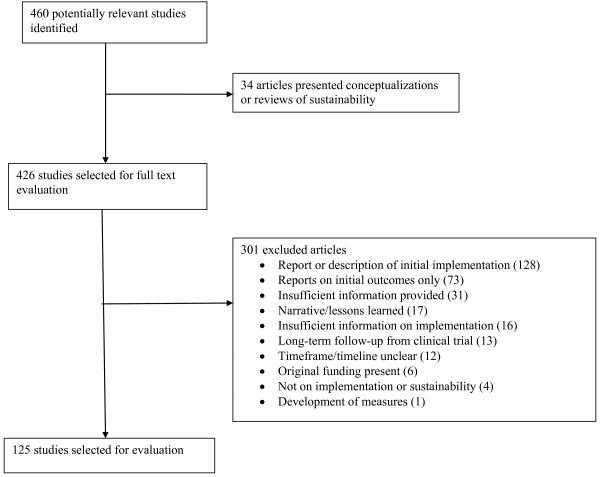
**Diagram of Study Selection and Exclusion Process**.

Figure 1 is a modified PRISMA (which stands for Preferred Reporting Items for Systematic Reviews and Meta-Analyses [33]) diagram summarizing the selection process, which includes reasons that potentially relevant papers were excluded. A total of 460 published articles were found and considered; 125 were determined to be relevant for coding (An additional file lists studies included in the review [see Additional file 1]). Of the papers included, 100 focused on or explicitly addressed sustainability. The remainder of the papers contained follow-up data on implementation from an intervention or training study, or focused primarily on dissemination or implementation but included information about sustainability.

### Coding

An initial coding scheme was developed based on constructs identified in previous conceptualizations of implementation [2-4,9,34,35] and sustainability [4,5,17,20,36-44]. Additional codes were generated deductively by the raters if a construct or process identified in the literature was not represented in the coding scheme. Related constructs among potential influences on sustainability were identified by consensus and collapsed under the general categories described in the findings. Thirty percent of the papers included in the review were coded by two raters and rater agreement was assessed. Agreement (Cohen's kappa) ranged from .85 to 1 ("substantial" to "almost perfect" [45]) on the broad categories and from .61 to 1 ("moderate" to "almost perfect" [45]) on more specific categories, which were later collapsed into the three broad categories. Additionally, disagreements on four items that were coded at lower frequencies with moderate agreement (.61-.80) were resolved by discussion and consultation if necessary with co-authors, resulting in consensus ratings. Two coders also rated 40% of the health-related (medical, public health/health promotion, or mental health) studies that reported sustainability outcomes for assessment method and the presence or absence of an indication of the level of quality or fidelity. Raters agreed on 93% of the ratings for assessment method and 90% for indication of quality or fidelity. The few disagreements were resolved through discussion.

## Results

### Characteristics of included studies

#### Area of study

Our search procedure identified studies from a variety of fields. Forty-one (33%) of the studies reported on medical interventions or healthcare programs, 42 (34%) on public health or health promotion programs, 33 (27%) on mental or behavioral health interventions, and 9 (7%) on educational interventions. Eighty-eight (72%) of the studies examined either programs or multicomponent interventions as opposed to a single procedure or intervention, such as a discrete medical procedure.

#### Unit of analysis

The majority of the studies (67; 54%) reported on sustainability at multiple implementation sites or settings, followed by studies that reported on sustainability at the individual or provider level (15; 12%). The remainder of the studies reported on sustainability within single systems or communities (20; 16%), at a single site (11; 9%), among individual providers within sites (7; 6%), or at the team level (5; 4%).

#### Timeframe examined

Studies were coded for the last post-implementation timeframe reported. Most studies (80; 64%) occurred two years or more past the initial implementation. Seven (6%) reported outcomes at less than 12 months post-implementation, 20 (16%) at 12 months, and 15 (12%) between 12 and 24 months post-implementation.

#### Terms and definitions

Sustainability was defined in a number of ways, and different terms were used to refer to the continuation of an innovation within an organization or community. Table 1 includes a listing of authors whose definitions were cited in the literature as working definitions of sustainability, as well as the frequency with which terms related to sustainability were used. Sixty-five percent of the studies examined did not present a definition. Among the studies that did present definitions, definitions were most commonly generated by the investigator. The most commonly used term in the studies examined was "sustainability," which was used in 62% of the articles. Those who cited a specific, published definition as their operational definition most frequently cited Scheirer's definition [25], which was based on the framework set forth by Shediac-Rizkallah and Bone [5], whose review was the second most commonly cited. Both identified multiple aspects of sustainability: continued benefits, continued activities, and continued capacity.

**Table 1:** Definitions of sustainability in reviewed studies

Focused on sustainability	N
Yes	102

No	23

**Defined sustainability**	**N**

Yes	36

No	80

Cited multiple definitions; didn't specify an operational definition	9

**Term used^a^:**	

Sustainability	77

Long-term/follow-up implementation	12

Institutionalization	6

Durability	3

Discontinuation	1

De-adoption	1

Maintenance	1

Sustained/continued implementation	1

Routinization	0

**Definition cited**	**N**

Other [9,32,46-56]^b^	12

Created definitions	8

Scheirer [25]	6

Shediac-Rizkallah and Bone [5]	4

Glasgow et al. [24]	2

Pluye et al. [57]	2

Goodman and Steckler [58]	2

^a^Some studies (e.g., follow-up studies from clinical trials) did not refer to sustainability or a related term; ^b^Each cited in one paper.

#### Methods used

Almost half of the studies reviewed employed self-report measures (54; 43%; nearly all were developed specifically for the project or study) or interviews (50; 40%) to assess sustainability or its influences. Fifty-four (43%) included some form of observation, 35 (28%) involved record review, and 23 (19%) included assessment of the fidelity/integrity of an intervention or practice. Eight (7%) of the studies reported on sustainability after an intervention had been implemented in a clinical trial. Solely quantitative approaches were used in 68 (54%) of the studies, qualitative approaches alone were used in 27 (22%), and 28 (23%) of the studies employed both qualitative and quantitative strategies. Nearly all examinations of sustainability were naturalistic rather than experimental. However, seven studies (6%) involved experimental manipulation of training or implementation strategies and assessed self-reported use, skill, or fidelity at a follow-up.

#### Outcomes reported

Studies discussed or reported on a variety of outcomes, and some reported multiple outcomes. Fifty-seven health-related studies (45% of the studies reviewed) reported outcomes such as the proportion of sites or providers sustaining, or the proportion of eligible patients receiving an intervention. The remaining studies did not report sustainability outcomes or reported data in such a way that it was not possible to determine the extent to which an intervention or practice was continued. For example, some reported on factors related to sustainability, without describing sustainability outcomes. Among the 57 studies that reported outcomes, 51 reported the proportion of sites or providers sustaining or discontinuing an intervention or program. The remaining studies reported the percent of patients or communities that received an intervention during a follow-up period. Seventy-five (60%) of the reviewed studies reported changes in the rate of program implementation and/or recipient outcomes, and two studies reported changes in both. Twenty-seven (22%) of the studies reported some form of health outcome (sustained impact or increases/decreases in desired outcomes), 14 of which were published in or after 2010.

#### Summary of findings

**Figure 2:**
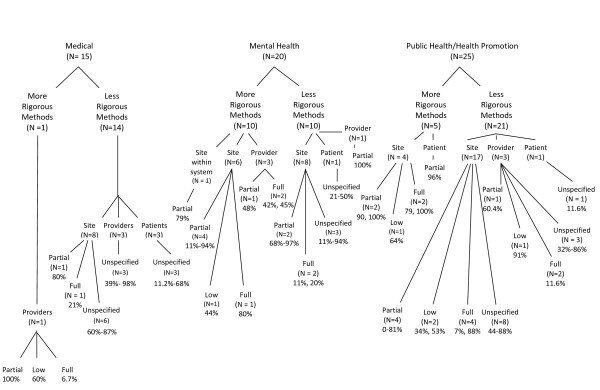
**Sustainability Outcomes By Field**. Note: More rigorous studies are defined as having included independent or objective observation and a judgment of fidelity, quality, or level of implementation. Ranges are provided when multiple studies reported these rates.

Figure 2 contains a summary of the sustainability outcomes reported for medical, public health/health promotion, and mental health studies. In general, a wide range of outcomes was reported. Rates of continuation of some, but not all, program or intervention elements ("partial sustainability") were relatively high across fields and units of analysis. Sixteen studies employed a form of independent observation and/or fidelity assessment to evaluate sustainability outcomes. In light of the literature that self-report assessments are often inaccurate [59], the figure distinguishes studies that employed observation from those that solely employed self-reports. Few studies that included independent observation or validation reported high rates of continuation at the site or setting level. The studies that reported on full sustainability or high fidelity at the provider level indicated that fewer than half of the observed providers sustained the practices at a high level of skill, intensity, or fidelity.

Of the 75 studies that reported on changes in implementation or recipient-level outcomes after initial implementation efforts or funding had ended, 56 studies reported on the intervention or program implementation. Of these, 19 reported lower levels of implementation after initial implementation efforts had ended, 17 reported an increase, and 3 reported no change or a similar level of implementation. Seventeen studies reported varying changes in rates across different intervention or program components. Twenty-one studies assessed changes in outcomes: 5 reported a decrease in desired outcomes, 10 reported an increase, and 1 reported no change. The remaining five studies reported multiple outcomes or indicators that varied in the extent to which they sustained.

#### Associated elements and influences

Thirty studies employed quantitative methodologies to identify predictors, correlates, or associated factors of sustainability. Thirty-six studies employed qualitative or mixed methodologies to identify influences on or processes associated with sustainability. Twenty of these studies specified that they were guided by a conceptual framework. Four broad categories of potential influences emerged in our coding process: influences related to the innovation, organizational context, capacity (internal and external), and processes. These four categories were common among each of the health-related fields we examined. However, only eight of the quantitative studies that examined elements related to sustainability included all four areas in their analyses, and 12 examined factors related to both the organization and capacity (typically, characteristics or attitudes of the workforce). Twelve of the studies, all of which employed qualitative or mixed methodologies, found that elements in all four categories were associated with sustainability.

Table 2 summarizes the findings, which are organized by study method (qualitative or quantitative) and health-related field (medicine/health care, public health/health promotion, and mental health). Findings regarding specific innovation characteristics and contextual factors were fairly consistent across medical/health care, public health/health promotion, and mental health studies. Findings related to capacity varied somewhat across fields. The presence of a champion was a less frequent finding for public health studies; funding was a much more common finding in this area. Workforce-related findings (e.g., adequate staffing, attributes of personnel) were less frequent findings in health care, and community support was less frequently identified as associated with the sustainment of mental health programs or interventions. Findings related to processes emerged most commonly in qualitative studies and were identified most commonly in public health programs. Perhaps due to the nature of the instruments or assessment procedures used in quantitative studies, processes were rarely identified. Engagement of stakeholders was more frequently associated with sustainability for public health studies, and adaptation of the intervention and alignment between the innovation and the setting were less frequently found in mental health studies.

**Table 2:** Influences on sustainability

	Overall			Health-related field-specific findings	
	**Number of quantitative findings** **(n = 30 studies)**	**Number of qualitative findings** **(n = 36 studies)**	**Number of medical intervention findings** **(n = 19)**	**Number of public health/health-promotion findings** **(n = 27)**	**Number of mental health findings** **(n = 22)**

**Innovation characteristics**	**11**	**18**	**7**	**12**	**10**
Fit	5	5	2	3	5
Ability to be modified/modifications made	4	7	2	5	4
Effectiveness or benefit	4	5	3	4	2
Ability to maintain fidelity/integrity	2	0	0	1	1
**Context**	**14**	**13**	**7**	**10**	**10**
Climate	0	2	1	0	1
Culture	2	1	2	1	0
Leadership	5	12	3	8	6
Setting characteristics (structure; policies)	11	2	4	4	5
System/policy change	2	5	3	3	1
**Capacity**	**15**	**23**	**11**	**14**	**12**
Champions (internal or external)	5	6	4	3	4
Funding	5	8	3	8	2
Workforce (staffing, attributes)	10	12	4	10	7
Resources	2	7	4	3	3
Community/stakeholder support/involvement	6	10	5	9	2
**Processes and interactions**	**8**	**27**	**10**	**16**	**8**
Engagement/relationship building	2	7	0	7	2
Shared decision making among stakeholders	3	2	2	2	1
Adaptation/alignment	2	5	2	5	0
Integration of rules/policies	3	10	4	6	2
Evaluation and feedback	2	6	1	4	2
Training and education	4	8	3	3	5
Collaboration/partnership	1	11	3	7	2
Navigating competing demands	0	4	1	2	1
Ongoing support	4	11	4	4	6
Planning	0	1	0	1	0

## Discussion

We examined 125 published papers to identify the different methodologies, types of innovations studied, timeframes examined, definitions used, outcomes examined, and factors examined in research on sustainability to date. Discussions in the literature over the past two decades [5,25,32] regarding the importance of sustainability appear to have resulted in an increase in research on this topic. However, our review found relatively few comprehensive or methodologically rigorous studies. The majority of the studies were retrospective. Most did not provide an operational definition of sustainability, and fewer than half appeared to be guided by a published definition or model of the concept. Few employed independent evaluation or observation.

### Sustainability outcomes

Because of the variety of results reported in the studies we reviewed, it is difficult to quantify or generalize about the extent to which new programs and practices are sustained. However, three findings are notable. First, similar to findings from a previous review [10], we found that those studies that provided information about levels or extent of implementation generally indicated that partial sustainability was more common than continuation of the entire program or intervention, even when full implementation was initially achieved. Most projects did not maintain all aspects as originally designed or implemented. However, in the studies that we reviewed, it was not possible to determine the impact of partially sustaining interventions on recipient-level outcomes. Further, virtually no studies revealed the nature of the changes made, the reasons for the changes, or the process by which adaptations or decisions to discontinue elements of the program or intervention were made. A second key finding is that in the past five years, there has been an increase in the number of studies that reported data on the sustainability of patient- or recipient-level benefits. Future studies that further examine these outcomes will be critical to understanding whether, and to what extent, the health-related benefits of implementation efforts can be sustained over time. Finally, the studies that employed independent fidelity ratings to assess sustainability at the provider level indicated that fewer than half of the providers sampled continued the practice or intervention at high levels of fidelity. These findings suggest that the development and study of fidelity-maintenance strategies, such as training and supervision, audit and feedback, building triggers into the process of care, checklists, or reminders, may be particularly important for the sustainment of interventions that require a high degree of fidelity to produce the intended health benefits [60-62].

### Influences on sustainability

Our review found that although terminologies and areas of emphasis differ somewhat across fields, influences on sustainability relate to the context (both outer, e.g., policies, legislation; and inner, e.g., culture, structure), the innovation itself (e.g., fit, adaptability, and effectiveness), processes (e.g., fidelity monitoring, evaluation, efforts to align the intervention and the setting), and the capacity to sustain (e.g., funding, resources, workforce characteristics and stability, interpersonal processes). Some qualitative findings also supported the conceptual literature that suggests an interrelation and interaction between these factors [63]. The broad categories of influences that our findings appeared to fit overlap most closely with the components of Shediac-Rizkallah and Bone's (1998) and Scheirers' (2005) conceptualizations, which were some of the more commonly cited definitions in the studies that we reviewed [5]. Within these broad categorizations, however, the key elements that were identified varied considerably.

Findings related to capacity were relatively common in both quantitative and qualitative research. For example, both qualitative and quantitative methodologies identified influences related to the workforce as associated with sustainability. These included the stability of the workforce and attributes of the workforce, such as their skills and attitudes. Additionally, qualitative studies identified the support or participation of key stakeholders and funding as important influences. Funding was rarely measured or included in the analyses, perhaps because studies took place after the initially allocated funding and resources had been removed. While some studies explicitly assessed or discussed the availability of new funds to support the programs that were being studied, most did not indicate whether additional funding had been obtained or allocated. However, influences such as sufficient resources and staffing that were identified in qualitative studies may be indicators of the adequacy of funds.

Other elements that are included in conceptualizations of sustainability [18] rarely emerged in the hypotheses or findings of the studies that we reviewed. Evaluation, feedback, and other quality-improvement processes were also less well represented than expected. Program or intervention effectiveness was identified in only nine studies, despite a fairly common emphasis on the importance of observable benefits within the implementation literature. In contrast to the relatively consistent emphasis on characteristics of the innovation within conceptualizations of sustainability and the broader implementation literature, fewer studies than expected found that characteristics of the innovation were associated with sustainability [3,9,64]. The dearth of findings related to innovation characteristics may be due to the lack of influence of the innovation on sustainability, but it may also be due to researchers' lack of attention to these constructs. Some researchers may have viewed innovation characteristics as more central to adoption decisions than to sustainment. Others may have overlooked innovation characteristics because they were examining a single innovation or organization and thus lacked sufficient variability to study the relative impact of factors such as fit or the intervention's complexity. Not surprisingly, among the innovation characteristics that were identified, the fit of the program or intervention with the system or organization and the degree to which the intervention or program could be modified were most common. Finally, given the amount of discussion on leadership, organizational climate, and culture in the literature on implementation and sustainability [5,20,35,39,65,66], we expected greater representation of these constructs in the studies that were reviewed.

Processes and interactions were associated with sustainability in nearly three-quarters of the qualitative studies. Integration of the program into policies, collaboration among stakeholders, and ongoing support were commonly identified processes. Findings related to processes that emerged in qualitative studies may explain why factors such as culture and climate were rarely identified in the studies we reviewed, despite their prominence in the implementation literature. Those who were interviewed in qualitative studies may have been more likely to describe noticeable processes and interactions that are evidence of a particular culture or leadership style than to characterize the culture of an organization. For example, some processes identified in our review, such as integration into policies or standards, may serve as "culture-embedding mechanisms" [67,68]. Similarly, negotiation, relationships, and shared decision making may be fostered by effective leaders and found more commonly in contexts that are prepared to nurture and sustain new practices. Studies with quantitative designs were less likely to identify processes and interactions of this nature, perhaps due to their design and research questions, as well as the models and measures that were employed. Important processes may have been subsumed, identified, or obscured under related and more readily measured constructs. The process-related findings highlight the importance of investigating the ways in which influences at multiple levels may interact to impact sustainability [19]. For example, some processes that were identified in our review suggest mutual adaptation between the intervention and the organization or system (e.g., adaptation of the intervention to improve fit, alignment of the organizational procedures with the intervention), or important interactions between stakeholders in various roles (e.g., negotiation, navigating competing demands). Such findings suggest that interplay between contextual factors and the innovation itself is to be expected given the dynamic nature of the complex systems into which innovations are introduced [19,69].

In summary, the findings that we presented above illustrate the variability in methods, outcomes, and potential influences that have been studied to date. Based on the empirical literature that we reviewed, it is difficult to generalize about influences on sustainability and the long-term impact of implementation efforts. As research develops further, refinements in conceptualizations and study designs will lead to results that are more easily interpreted. Below, we discuss some considerations and recommendations for such research.

### Recommendations for advancing the empirical literature

#### Defining sustainability

An important limitation to the body of research on sustainability that we reviewed is the high proportion of studies that did not present a working definition or demonstrate evidence of guidance by a model of sustainability. The way that the concept is defined and conceptualized has important implications for how it is investigated. At a basic level, the studies that we reviewed focused on the continuation of the programs and practices that were implemented within organizations, systems, or communities after initial implementation efforts or funding ended [70]. While such a broad definition applies across a number of disciplines and contexts, research based on such a definition can yield results that are difficult to interpret, particularly when the studies conclude that some aspects of a program or innovation continued while others did not. Thus, we recommend that both a definition and a conceptual framework be carefully chosen to guide research in this area.

In light of our review, we suggest that investigators consider several factors in choosing a definition to guide their research on the sustainment of interventions or programs and that they clearly specify their research questions regarding each factor. These factors are (1) whether, and to what extent, the core elements (the elements most closely associated with desired health benefits) [23,32,64] are maintained; (2) the extent to which desired health benefits are maintained or improved upon over time after initial funding or supports have been withdrawn; (3) the extent, nature, and impact of modifications to the core and adaptable/peripheral elements of the program or innovation [23,32]; and (4) continued capacity to function at the required level to maintain the desired benefits. A program or intervention's *impact *may be considered sustained if desired health benefits remain at or above the level achieved during implementation and this increase can be attributed to continuation of the program. A program or intervention may be considered to be sustained at a given point in time if, after initial implementation support has been withdrawn, core elements are maintained (e.g., remain recognizable [13] or delivered at a sufficient level of fidelity or intensity to yield desired health outcomes [59,62,71]) and adequate capacity for continuation of these elements is maintained.

#### Defining outcomes or desired benefits

As our discussion of elements of sustainability above indicates, the desired impact and benefits of the program or intervention should be identified. Additionally, stakeholder goals for sustainability (e.g., Must the program be sustained at the same level, or improved upon? To what extent is a lower level of implementation fidelity or a partially sustained program consistent with stakeholders' goals for the project? At what point, and under what circumstances, is discontinuation, modification, or implementation of a more effective, efficient, or better-fitting intervention advisable?) should be considered in the interpretation of findings. The type of innovation and setting will drive some of these considerations. For specific interventions identified to improve patient-level outcomes (e.g., reduce rates of infection, relieve symptoms), these health benefits may be considered to be the "bottom line." For programs formed to identify and implement multiple interventions to achieve health-related goals, outcomes such as indicators that programs are being implemented, the existence and functioning of a decision-making body, and coordination between multiple agencies or stakeholders may be critical outcomes in addition to population-level outcomes such as reduced rates of disease or infection.

#### Choosing an appropriate timeframe

When studying the sustainment of a program or intervention, a timeframe that is sufficiently beyond an initial implementation effort to provide meaningful information must be chosen. Although there may be no obvious indicator for determining when initial implementation efforts have "ended," it can be useful conceptually to separate the period of initial implementation from a post-implementation phase. Most of the studies we reviewed examined sustainability two or more years after implementation, consistent with suggestions in the literature [24]. Although many existing conceptualizations imply that sustainability cannot be studied until full implementation is achieved [31] and funding is withdrawn [4], some programs may never be fully implemented due to a variety of forces within or external to systems and organizations. For example, some may have funding withdrawn before full implementation is achieved [5], yet these programs may achieve success in maintaining some components of the programs over time. Additionally, as most studies that we reviewed measured sustainability at a single time point, they may have masked what several conceptualizations present as a dynamic phenomenon. To advance what is currently known about sustainability over time and to capture variations over time, we suggest that researchers assess sustainability over several years rather than at a single time.

#### Studying fidelity and adaptation

Consistent with discussions of sustainability that suggest that adaptation and evolution of the practices and innovations are to be expected [25,32,72,73], a number of studies that we reviewed indicated that some form of modification had occurred. While such changes may be made to interventions or programs in response to contextual influences, such as shifting priorities or availability of resources, the process and nature of adaptations may vary considerably between projects. Most studies that we reviewed did not describe adaptations or examine their impact on health-related outcomes. To facilitate a greater understanding through future research, some clarity regarding adaptation and fidelity is necessary. Additional research is needed to assess the conditions under which fidelity, or different types and degrees of adaptations, are important for the achievement of specific health benefits. While it is important to differentiate sustainment from entrenchment, which may *prevent *further innovation or adoption of more effective practices [16,31,32], it is also critical to understand when, and to what components of a particular program or intervention, fidelity is necessary. Fidelity has been conceptualized in the mental health literature as a combination of adherence to a prescribed set of practices at adequate dose or intensity, competence in delivery, and differentiation from other interventions [59,74], with judgments of competence taking response to certain contextual factors into account [23]. In the medical literature, it has been defined as "the extent to which the system provides patients the precise interventions they need, delivered properly, precisely when they need them" [71]. Evidence has emerged that for some interventions, a higher level of fidelity or intensity may be required to produce desired health benefits [11,62,74]. In these cases, insufficient levels of fidelity may in fact indicate that a program was not sustained at the level necessary to promote these outcomes. On the other hand, the success of some programs (e.g., community-based health promotion programs) may be less dependent on the implementation of a set of procedures with fidelity than on the flexibility and adaptive capacity of the system or organization that implements the program. In such cases, the range of possible or even necessary adaptations within the program might be quite broad [75,76] and may reflect new priorities or response to local conditions [77]. This type of ongoing evaluation, modification, and replacement of elements or procedures as necessary is an approach advocated in organizational learning and continuous quality-improvement literatures [78-80]. Theory in this area suggests that an appropriate balance between exploration of new methods while exploiting existing knowledge regarding effective strategies may in fact result in more sustainable and successful programs [81,82].

Simply measuring fidelity and characterizing modifications as deviations may obscure the very refinements that facilitate the continued use of some innovations. A period of mutual adaptation [83] is probably common between initial implementation and institutionalization, and some innovations may continually evolve [76]. To advance the field, subsequent research should include further attention to the nature of the modifications that occur and the process by which modifications are made [84,85]. Even for those interventions for which there is evidence that fidelity is important, there may be aspects that can be adapted and modified, while preserving desired outcomes [23,86,87], provided that the critical elements are conducted or delivered at adequate levels of fidelity. Several types of modification, at either a molecular or molar level [74,88], may occur as practitioners, communities, and systems implement specific programs and interventions. For example, tailored adaptation may be guided by available evidence and remain faithful to identified core elements [23], with an eye towards facilitating desired health benefits. Evolution may occur if procedures are modified in light of the emergence of new evidence [89]. Replacement may occur if more compatible or effective interventions or procedures are identified [9]. Adaptations that result in reversion or erosion fail to preserve the core elements of an intervention, which may in turn result in a failure to preserve desired health outcomes. In such cases, if the intervention that was originally introduced becomes unrecognizable [13], it may be considered to have been discontinued. We therefore recommend that when the intervention is the focal unit of interest, in addition to identifying methods of assessing fidelity, researchers study periods of adaptation [90] and characterize the nature of modifications made to interventions. It will also be important to understand more about the nature of possible trade-offs that are made between fidelity and sustainability and how stakeholders make such decisions.

Identifying core elements, or components that are critical for the achievement of desired outcomes, is also a critical area for future study. Developers of many complex interventions have not yet pursued these questions. Isolating elements of innovative practices and examining their relative contributions to the overall impact of the practice can be challenging and may not be feasible or desirable in some situations. However, when available, this information can facilitate a streamlined or pragmatic implementation effort that retains the aspects found to be most effective and successful in everyday practice [16]. As a positive impact on intended recipients is the ultimate goal of implementation, we recommend that researchers include a consideration of these important matters in their efforts to study sustainability.

#### Conceptualization, measurement, and assessment of influences on sustainability

Findings from our review suggest that the study of influences on sustainability is nascent. Fewer studies than we expected identified influences that are found in existing conceptualizations of sustainability. It is possible that these findings result from a lack of guidance by a theoretical framework, given that fewer than one-third of the studies that we reviewed were guided by an explicit model. To advance research in this area, we recommend that researchers identify models or frameworks of sustainability [91] that are most appropriate for their projects and research questions. In doing so, consideration should be given to the issues regarding fidelity, the potential for adaptation, and the nature of the system that will be studied. As many models of sustainability have not been evaluated [19,20,92], we do not yet know enough about which models are valid and appropriate [93] for differing programs and circumstances [16]. Thus, efforts to evaluate conceptualizations of sustainability can further advance the field.

There is also room for improvement in methods employed to characterize intervention sustainability and its influences. Beyond fidelity measures used in clinical trials, there are few procedures or benchmarks to guide researchers in efforts to identify the extent to which interventions and programs were continued as implemented. Pluye and colleagues operationalized definitions of three degrees of sustainability for public health programs (weak, moderate, and high) but did not develop a formal assessment instrument. However, they did develop a 15-question interview to assess degrees of sustainability [41]. The Level of Institutionalization scale has been developed to gauge the extent to which key activities for a health-promotion program have occurred [94,95], and the authors suggest that the measure can be modified easily for a variety of health-promotion programs and settings. When fidelity is necessary to sustain outcomes, observation using a set of criteria for adequate skill, adherence, or intensity will improve the precision with which results are reported. As in other areas, self-reports of fidelity are likely to be imprecise [59]. The development of valid, yet low time- and cost-intensive, observation or monitoring strategies would represent a significant advance [59]. Triangulation of information gathered through multiple methods may ultimately be most informative. As fidelity measures are generally not designed to assess, describe, or elucidate the nature and consequences of adaptations, methods of assessment in this area must also be advanced [72].

Typically, in research that employs surveys to measure influences on sustainment, the instruments were developed for the specific projects or implementation efforts [40], and psychometric properties were almost never reported. The development of a wholly unique procedure for assessing the sustainability of each intervention or program limits the conclusions that can be drawn from the literature as a whole. In lieu of specific measures, other studies employed survey results or information about setting characteristics collected during the implementation process to identify predictors [c.f.96]. Thus, assessment and analytic strategies employed to date may not have captured the appropriate influences and their interactions. Multilevel measurement of sustainability [20,29], based on sound conceptualization, is necessary to allow for greater methodological rigor and interpretability of findings [91], and some measures have been developed for this purpose. Mancini and Marek developed a 29-item Program Sustainability Index to assess six factors related to the sustainability of community-based programs [40]. An instrument was also developed based on the National Health Service Institute for Innovation and Improvement's Health Service Sustainability model. While the model was intended to be used in the planning and early stages of implementation to evaluate the likelihood that an innovation will be sustained, the authors suggest that it can be used at any phase of a project [13]. Both of these surveys assess factors and processes at multiple levels and can be used to examine the relationship and interactions among differing elements and levels, although further research on their validity and applicability to a broad range of programs or interventions is warranted.

Qualitative and mixed methodologies that assess potential influences across multiple levels will continue to be necessary to refine hypotheses, explore results, understand the relationships between sustainability drivers, and facilitate the development of interventions to promote the sustainability of effective programs and practices. Our review indicates that qualitative studies yielded a wider variety of findings and have highlighted processes and constructs that warrant further study. However, the vast majority did not provide interview guides to clarify how they assessed activities, processes, or influences associated with sustainability. This step will improve the interpretability and replicability of future research. Furthermore, prospective research on sustainability and efforts to identify influences and interventions that lead to sustainable implementation efforts will be of critical importance [23]. Elucidating the ways in which influences interact to enhance or challenge sustainability will ultimately facilitate an understanding from a complex-systems perspective and may also lead to the development of strategies to promote sustainability in contexts and circumstances in which certain factors are absent or less than optimal. For example, whether specific influences (e.g., leadership, culture) or processes can serve as protective or compensatory factors in the absence of other elements (e.g., funding) remains to be determined. Findings in this area can ultimately lead to the development and improvement of strategies that promote the continuation of effective programs and interventions.

### Limitations

Some limitations to our review are important to acknowledge. In this project, we reviewed studies that identified specific programs or interventions and investigated either the extent to which those interventions and/or the desired benefits were sustained or factors that influenced their sustainability. Information generated from this review can inform researchers about what has not yet been sufficiently explored and stakeholders about what may be important to consider when monitoring specific programs or interventions that they have chosen to implement. We did not specifically seek studies that examined the adaptive capacity of systems, and we did not take an ecological or developmental perspective in our review [19,97]. Such perspectives are valuable for future research and much can be learned by broadening the research questions beyond whether or not an intervention continued as originally implemented. However, from a number of stakeholder perspectives, and given the substantial resources that have been devoted to implementing effective practices to date, there is also value to understanding the findings and limitations of the existing body of research that has investigated whether and how interventions and their health benefits have been sustained [16]. Thus, in this review, the ways in which we presented our findings, conclusions that we drew, and recommendations that we made were shaped by an effort to understand more about sustainment or discontinuation from this perspective and by the state of the existing literature that has addressed sustainability in this manner.

Although we attempted to identify studies from a variety of fields using a number of search strategies, the diffuse nature of the literature on sustainability and the variety of terms used may have limited our ability to complete an exhaustive review. Additionally, we sought to look broadly across literatures from a number of fields, but the applicability of some findings to any one innovation may be somewhat limited. We sought to learn what the available findings could tell us about the extent to which specific practices or programs have become rooted and sustained within organizations and communities, in order to conduct the most comprehensive review possible. By "casting a broad net" in terms of the fields and methodologies that were represented in our review, we intended to identify methods, strategies, constructs, and findings that may not have been considered within some individual fields. In these studies, the extent to which a program or intervention had continued was generally assessed at a single point in time, limiting conclusions that could be drawn about changes over time. Thus, we chose to present ranges of sustainment that had been found within particular fields in lieu of a definitive statement about whether or to what extent sustainment could be expected for particular innovations.

## Conclusions

In the early efforts to study the sustainability of specific programs and interventions that we reviewed, we have identified a body of literature that is fragmented and underdeveloped. In addition to previously noted challenges, limited funding for monitoring programs after initial implementation, challenges to observation in real-time, and the lack of validated measures have complicated the study of sustainability, and much of what is known to date has been determined through *post hoc *research [4]. The current paper contributes to the literature by reviewing the research on sustainability that has been conducted to date. Our goals in this review were to examine the ways that researchers have approached this challenging topic thus far and to contribute to the development of an agenda for future, high-quality research.

Each of our recommendations to advance what is known about sustainability will require time, resources, and funding, all of which have been relatively limited across the fields that we reviewed. With prospective studies of implementation efforts underway, investigators could make a substantial contribution to the field by planning follow-up studies that assess the degree to which the programs or practices are maintained and the nature and implications of changes that are made once implemented. Furthermore, researchers and policy makers should be encouraged to consider the question of sustainability when developing implementation programs and research. Appropriate planning, assessment, and allocation of funds would result in much better understanding of why and how some interventions and programs last and others do not. In an era of increasing budget pressures and greater scrutiny of new investments, nothing could be more desirable as a practical matter as well.

## Competing interests

The authors declare that they have no competing interests.

## Authors' contributions

SWS conceptualized the study, contributed to the data collection and coding, and was the predominant contributor to this article. NC and AC assisted with the coding of articles and compilation and interpretation of results. MC, JK, and FC made significant contributions to the conceptual framework and the interpretation of results. All authors read and modified drafts and approved the final manuscript.

## Supplementary Material

Additional file 1 **Articles Included in the Review **[37,54,98-220]. Click here for file
