# Supplementary material for: The sustainability of new programs and innovations: a review of the empirical literature and recommendations for future research
Source: Implement Sci. 2012 Mar 14;7:17. doi: 10.1186/1748-5908-7-17 (PMC3317864; doi:10.1186/1748-5908-7-17)
Supplement: Additional file 1 — Articles Included in the Review [37,54,98-220]. [file 1748-5908-7-17-S1.DOC]

**Articles included in our review**

**1.** Abraham AJ, Knudsen HK, Roman PM: **A longitudinal examination of alcohol pharmacotherapy adoption in substance use disorder treatment programs: patterns of sustainability and discontinuation**. *J Stud Alcohol Drugs* 2011, **72**(4):669-677.

**2.** Ahluwalia IB, Robinson D, Vallely L, Gieseker KE, Kabakama A: **Sustainability of community-capacity to promote safer motherhood in northwestern Tanzania: what remains?** *Global Health Promotion* 2010, **17**(1):39-49.

**3.** Aitaoto N, Tsark J, Braun KL: **Sustainability of the Pacific Diabetes Today coalitions.** *Prev Chronic Dis* 2009, **6**(4):A130-A138.

**4.** Amazigo U, Okeibunor J, Matovu V, Zouré H, Bump J, Seketeli A: **Performance of predictors: evaluating sustainability in community-directed treatment projects of the African programme for onchocerciasis control.** *Soc Sci Med* 2007, **64**(10):2070-2082.

**5.** August GJ, Bloomquist ML, Lee SS, Realmuto GM, Hektner JM: **Can evidence-based prevention programs be sustained in community practice settings? The early risers' advanced-stage effectiveness trial.** *Prev Sci* 2006, **7**(2):151-165.

**6.** Austin G, Bell T, Caperchione C, Mummery WK: **Translating research to practice: using the RE-AIM framework to examine an evidence-based physical activity intervention in primary school settings.** *Health Promotion Practice* 2011, **12**(6):932-941.

**7.** Babl FE, Krieser D, Belousoff J, Theophilos T: **Evaluation of a paediatric procedural sedation training and credentialing programme: sustainability of change.** *Emergency Medical Journal* 2010, **27**(8):577-581.

**8.** Baer JS, Ball SA, Campbell BK, Miele GM, Schoener EP, Tracy K: **Training and fidelity monitoring of behavioral interventions in multi-site addictions research.** *Drug Alcohol Depend* 2007, **87**(2):107-118.

**9.** Bailie RS, Robinson G, Kondalsamy-Chennakesavan SN, Halpin S, Wang Z: **Investigating the sustainability of outcomes in a chronic disease treatment programme.** *Soc Sci Med* 2006, **63**(6):1661-1670.

**10.** Barnett LM, Van Beurden E, Eakin EG, Beard J, Dietrich U, Newman B: **Program sustainability of a community-based intervention to prevent falls among older Australians.** *Health Promot Int* 2004, **19**(3):281-288.

**11.** Baum F, Jolley G, Hicks R, Saint K, Parker S: **What makes for sustainable Healthy Cities initiatives?--a review of the evidence from Noarlunga, Australia after 18 years.** *Health Promot Int* 2006, **21**(4):259-265.

**12.** Beery WL, Senter S, Cheadle A, Greenwald HP, Pearson D, Brousseau R, Nelson GD: **Evaluating the legacy of community health initiatives: a conceptual framework and example from the California Wellness Foundation's health improvement initiative.** *Am J Evaluation* 2005, **26**:150-165.

**13.** Bere E, Veierod MB, Bjelland M, Klepp KI: **Free school fruit--sustained effect 1 year later.** *Health Educ Res* 2006, **21**(2):268-275.

**14.** Bisset S, Potvin L: **Expanding our conceptualization of program implementation: lessons from the genealogy of a school-based nutrition program.** *Health Educ Res* 2006, **22**(5):737-746.

**15.** Blasinsky M, Goldman HH, Unutzer J: **Project IMPACT: a report on barriers and facilitators to sustainability.** *Adm Policy Ment Health* 2006, **33**(6):718-729.

**16.** Bowman C, Sobo E, Asch S, Gifford A, & The H. I. V. Hepatitis Quality Enhancement Research Initiative: **Measuring persistence of implementation: QUERI series.** *Implementation Science* 2008, **3**(1):21.

**17.** Bratcht NF, Finnegan JR, Rissel C, Weisbrod R, Gleason J, Corbett J, Mortenson S: **Community ownership and program coninuation following a health demonstration program.** *Health Education Research* 1994, **9**:243-255.

**18.** Brand C, Landgren F, Hutchinson A, Jones C, MacGregor L, Campbell D: **Clinical practice guidelines: barriers to durability after effective early implementation.** *Intern Med J* 2005, **35**(3):162-169.

**19.** Bunik M, Federico MJ, Beaty B, Rannie M, Olin JT, Kempe A: **Quality improvement for asthma care within a hospital-based teaching clinic.** *Academic Pediatrics* 2011, **11**(1):58-65.

**20.** Cherry RA, West CE, Hamilton MC, Rafferty CM, Hollenbeak CS, Caputo GM: **Reduction of central venous catheter associated blood stream infections following implementation of a resident oversight and credentialing policy.** *Patient Safety in Surgery* 2011, **5**:15.

**21.** DeWein M, Miller L: **The effects of a classroom-based intervention on aggression-related injuries.** *Child and Youth Care Forum* 2009, **38**(4):201-218.

**22.** Dückers MLA, Wagner C, Vos L, Groenewegen PP: **Understanding organisational development, sustainability, and diffusion of innovations within hospitals participating in a multilevel quality collaborative.** *Implementation Science* 2011, **6**(1):18.

**23.** Ebert-May D, Derting TL, Hodder J, Momsen JL, Long TM, Jardeleza SE: **What we say is not what we do: effective evaluation of faculty professional development programs.** *BioScience* 2011, **61**(7):550-558.

**24.** Edvarsson K, Garvare R, Ivarsson A, Eurenius E, Morgen I, Nystrom M: **Sustainable practice change: professionals' experiences with a multisectoral child health promotion programme in Sweden.** *BMC Health Services Research* 2011, **11**:61.

**25.** Eliason RN: **Towards sustainability in village health care in rural Cameroon.** *Health Promot Int* 1999, **14**:301-306.

**26.** Ellingson K, Muder RR, Jain R, Kleinbaum D, Feng PJ, Cunningham C, Squier C, Lloyd J, Edwards J, Gebski V, Jernigan J: **Sustained reduction in the clinical incidence of methicillin-resistant *Staphylococcus aureus* colonization or infection associated with a multifaceted infection control intervention.** *Infection Control and Hospital Epidemiology* 2011***,* 32**(1):1-8.

**27.** Epstein JN, Langberg JM, Lichtenstein PK, Kolb RC, Stark LJ: **Sustained improvement in pediatricians' ADHD practice behaviors in the context of a community-based quality improvement initiative.** *Children's Health Care* 2010, **39**(4):296-311.

**28.** Escoffery C, Glanz K, Hall D, Elliott T: **A multi-method process evaluation for a skin cancer prevention diffusion trial.** *Eval Health Prof* 2009, **32**(2):184-203.

**29.** Evashwick C, Ory M: **Organizational characteristics of successful innovative health care programs sustained over time.** *Fam Community Health* 2003, **26**(3):177-193.

**30.** Feinberg ME, Bontempo DE, Greenberg MT: **Predictors and level of sustainability of community prevention coalitions.** *Am J Prev Med* 2008, **34**(6):495-501.

**31.** Fonck K, Claeys P, Bashir F, Bwayo J, Fransen L, Temmerman M: **Syphilis control during pregnancy: effectiveness and sustainability of a decentralized program.** *Am J Public Health* 2001, **91**(5):705-707.

**32.** Glaser EM: **Durability of innovations in human service organizations.** *Science Communication* 1981, **3**(2):167-185.

**33.** Glaser EM, Backer TE: **Durability of innovations: how goal attainment scaling programs fare over time.** *Community Ment Health J* 1980, **16**(2):130-143.

**34.** Glisson C, Schoenwald SK, Kelleher K, Landsverk J, Hoagwood KE, Mayberg S, Green P: **Therapist turnover and new program sustainability in mental health clinics as a function of organizational culture, climate, and service structure.** *Adm Policy Ment Health* 2008, **35**(1-2):124-133.

**35.** Godley SH, Garner BR, Smith JE, Meyers RJ, Godley MD: **A large scale dissemination and implementation model for evidence based treatment and continuing care.** *Clinical Psychology: Science and Practice* 2011*,* **18**(1):67-83.

**36.** Goetz MB, Hoang T, Henry SR, Knapp H, Anaya HD, Gifford AL, Asch SM: **Evaluation of the sustainability of an intervention to increase HIV testing.** *J Gen Intern Med* 2009, **24**(12):1275-1280.

**37.** Goodson P, Murphy Smith M, Evans A, Meyer B, Gottlieb NH: **Maintaining prevention in practice: survival of PPIP in primary care settings.** *Am J Prev Med* 2001, **20**(3):184-189.

**38.** Greenwood CR, Tapia Y, Abbott M, Walton C: **A building-based case study of evidence-based literacy practices: implementation, reading behavior, and growth in reading fluency, K-4.** *The Journal of Special Education* 2003, **37**(2):95-110.

**39.** Gruen RL, Elliott JH, Nolan ML, Lawton PD, Parkhill A, McLaren CJ, Lavis JN: **Sustainability science: an integrated approach for health-programme planning.** *The Lancet* 2008, **372**(9649):1579-1589.

**40.** Gurtler RE, Kitron U, Cecere MC, Segura EL, Cohen JE: **Sustainable vector control and management of Chagas disease in the Gran Chaco, Argentina.** *Proceedings of the National Academy of Sciences of the United States of America* 2007, **104**(41):16194-16199.

**41.** Harris DL, Henry RC, Bland CJ, Starnaman SM, Voytek KL: **Lessons learned from implementing multidisciplinary health professions educational models in community settings.** *J Interprof Care* 2003, **17**(1):7-20.

**42.** Helfrich CD, Damschroder LJ, Hagedorn HJ, Daggett GS, Sahay A, Ritchie M, Damush T, Guihan M, Ullrich PM, Stetler CB: **A critical synthesis of literature on the promoting action on research implementation in health services (PARIHS) framework.** *Implementation Science* 2010, **5**:82.

**43.** Henggeler SW, Sheidow AJ, Cunningham PB, Donohue BC, Ford JD: **Promoting the implementation of an evidence-based intervention for adolescent marijuana abuse in community settings: testing the use of intensive quality assurance.** *J Clin Child Adolesc Psychol* 2008, **37**(3):682-689.

**44.** Higuchi KS, Davies BL, Edwards N, Ploeg J, Virani T: **Implementation of clinical guidelines for adults with asthma and diabetes: a three-year follow-up evaluation of nursing care.** *Journal of Clinical Nursing* 2011, **20**(9-10):1329-1338.

**45.** Hii JL, Chee KC, Vun YS, Awang J, Chin KH, Kan SK: **Sustainability of a successful malaria surveillance and treatment program in a Runggus community in Sabah, east Malaysia.** *The Southeast Asian Journal of Tropical Medicine and Public Health* 1996, **27**(3):512-521.

**46.** Hoelscher DM, Feldman HA, Johnson CC, Lytle LA, Osganian SK, Parcel GS, Kelder SH, Stone EJ, Nader PR: **School-based health education programs can be maintained over time: results from the CATCH Institutionalization study.** *Prev Med* 2004, **38**(5):594-606.

**47.** Hoffman KA, Ford II, James H, Choi D, Gustafson DH, McCarty D: **Replication and sustainability of improved access and retention within the Network for the Improvement of Addiction Treatment.** *Drug Alcohol Depend* 2008, **98**(1-2):63-69.

**48.** Hogg W, Baskerville N, Nykiforuk C, Mallen D: **Improved preventive care in family practices with outreach facilitation: understanding success and failure.** *J Health Serv Res Policy* 2002, **7**(4):195-201.

**49.** Hoque BA, Juncker T, Sack RB, Ali M, Aziz KM: **Sustainability of a water, sanitation and hygiene education project in rural Bangladesh: a 5-year follow-up.** *Bulletin WHO* 1996, **74**(4):431-437.

**50.** Jansen M, Harting J, Ebben N, Kroon B, Stappers J, VanEngelshoven E, deVries, N: **The concept of sustainability and the use of outcome indicators: a case study to continue a successful health counselling intervention.** *Fam Pract* 2008, **25**:i32–i37.

**51.** Johnson C, Fargo J, Kahle JB: **The cumulative and residual impact of a systemic reform program on teacher change and student learning of science.** *School Science and Mathematics* 2010*,* **110**(3):144-159.

**52.** Kalafat J, Ryerson DM: **The implementation and institutionalization of a school-based youth suicide prevention program.** *The Journal of Primary Prevention* 1999, **19**(3):157-175.

**53.** Kay BH, Tuyet Hanh TT, Le NH, Quy TM, Nam VS, Hang PV, Yen NT, Hill PS, Vos T, Ryan PA: **Sustainability and cost of a community-based strategy against *Aedes aegypti* in northern and central Vietnam.** *The American Journal of Tropical Medicine and Hygiene* 2010, **82**(5):822.

**54.** Kellie SM, Timmins A, Brown C: **A statewide collaborative to reduce methicillin-resistant *Staphylococcus aureus* bacteremias in New Mexico.** *Joint Commission Journal on Quality and Patient Safety* 2011, **37**:154-162.

**55.** Kennedy MT, Fiss PC: **Institutionalization, framing, and diffusion: the logic of TQM adoption and implementation decisions among U.S. hospitals.** *Acad Manage J* 2009, **52**(5):897-918.

**56.** Klingner J, Vaughn S, Tejero Hughes M, Arguelles ME: **Sustaining research-based practices in reading: a 3-year follow-up.** *Rem Spec Ed* 1999, **20**(5):263-275.

**57.** Knapp H, Anaya HD, Goetz MB: **Attributes of an independently self-sustaining implementation: nurse-administered HIV rapid testing in VA primary care.** *Quality Management in Healthcare* 2010*,* **19**(4):292-297.

**58.** Knippenberg R, Soucat A, Oyegbite K, Sene M, Bround D, Pangu K, Hopwood I, Grandcourt R, Tinguiri KL, Fall I, Ammassari S, Alihonou E: **Sustainability of primary health care including expanded program of immunizations in Bamako Initiative programs in West Africa: an assessment of 5 years' field experience in Benin and Guinea.** *International Journal of Health Planning and Management* 1997, **12**:S9-S28.

**59.** Knudsen HK, Studts JL: **Availability of nicotine replacement therapy in substance use disorder treatment: longitudinal patterns of adoption, sustainability, and discontinuation.** *Drug Alcohol Depend* 2011, **118**(2-3):244-250.

**60.** Kolko DJ, Iselin A-MR, Gully KJ: **Evaluation of the sustainability and clinical outcome of Alternatives for Families: A Cognitive-Behavioral Therapy (AF-CBT) in a child protection center.** *Child Abuse & Neglect* 2011, **35**(2):105-116.

**61.** Lafond AK: **Improving the quality of investment in health: lessons on sustainability.** *Health Policy and Planning* 1995, **10**:63-76.

**62.** LaPelle NR, Zapka J, Ockene JK: **Sustainability of public health programs: the example of tobacco treatment services in Massachusetts.** *Am J Public Health* 2006, **96**:1363-1369.

**63.** Lee AJ, Bonson APV, Yarmirr D, O'Dea K, Mathews JD: **Sustainability of a successful health and nutrition program in a remote Aboriginal community.** *The Medical Journal of Australia* 1995, **162:**632-635.

**64.** Lee PW, Dietrich AJ, Oxman TE, Williams JWJ, Barry SL: **Sustainable impact of a primary care depression intervention.** *Journal of the American Board of Family Medicine* 2007, **20**(5):427-433.

**65.** Lichtenstein E, Thompson B, Nettekoven L, Corbett K: **Durability of tobacco control activities in 11 North American communities: life after the community intervention trial for smoking cessation (COMMIT).** *Health Educ Res* 1996, **11**(4):527-534.

**66.** Lieber J, Butera G, Hanson M, Palmer S, Horn E, Czaja C: **Sustainability of a preschool curriculum: what encourages continued use among teachers?** *NHSA Dialog* 2010, **13**(4):225-242.

**67.** Lodl K, Stevens G: **Coalition sustainability: long-term successes & lessons learned.** *Journal of Extension* 2002, **40**(1):1-8.

**68.** Loman SL, Rodriguez BJ, Horner RH: **Sustainability of a targeted intervention package: first step to success in Oregon.** *Journal of Emotional and Behavioral Disorders* 2010, **18**(3):178-191.

**69.** Lyon AR, Stirman SW, Kerns SE, Bruns EJ: **Developing the mental health workforce: review and application of training approaches from multiple disciplines.** *Adm Policy Ment Health* 2011, **38**(4):238-253.

**70.** Martin GW, Herie MA, Turner BJ, Cunningham JA: **A social marketing model for disseminating research-based treatments to addictions treatment providers.** *Addiction* 1998, **93**(11):1703-1715.

**71.** Massatti RR, Sweeney HA, Panzano PC, Roth D: **The de-adoption of innovative mental health practices (IMHP): why organizations choose not to sustain an IMHP.** *Adm Policy Ment Health* 2008, **35**(1-2):50-65.

**72.** Maticka-Tyndale E, Wildish J, Gichuru M: **Thirty-month quasi-experimental evaluation follow-up of a national primary school HIV intervention in Kenya.** *Sex Education: Sexuality, Society and Learning* 2010, **10**(2):113-130.

**73.** Mayer J, Mooney B, Gundlapalli A, Harbarth S, Stoddard GJ, Rubin MA, Eutropius L, Brinton B, Samore MH: **Dissemination and sustainability of a hospital-wide hand hygiene program emphasizing positive reinforcement.** *Infection Control and Hospital Epidemiology* 2011, **32**(1):59-66.

**74.** McCormick LK, Steckler AB, McLeroy KR: **Diffusion of innovations in schools: a study of adoption and implementation of school-based tobacco prevention curricula.** *Am J Health Promot* 1995, **9**(3):210-219.

**75.** McDermott R, Tulip F, Schmidt B, Sinha A: **Sustaining better diabetes care in remote indigenous Australian communities.** *Quality and Safety in Health Care* 2004, **13**(4):295-298.

**76.** McHugo GJ, Drake RE, Whitley R, Bond GR, Campbell K, Rapp CA, Goldman HH, Lutz WJ, Finnerty MT: **Fidelity outcomes in the national implementing evidence-based practices project.** *Psychiatr Serv* 2007, **58**(10):1279-1284.

**77.** Miller WR, Yahne CE, Moyers TB, Martinez J, Pirritano M: **A randomized trial of methods to help clinicians learn motivational interviewing.** *J Consult Clin Psychol* 2004, **72**(6):1050-1062.

**78.** Morgenstern LB, Bartholomew LK, Grotta JC, Staub L, King M, Chan W: **Sustained benefit of a community and professional intervention to increase acute stroke therapy.** *Arch Intern Med* 2003, **163**(18):2198-2202.

**79.** Nease Jr DE, Nutting PA, Graham DG, Dickinson WP, Gallagher KM, Jeffcott-Pera M: **Sustainability of depression care improvements: success of a practice change improvement collaborative.** *The Journal of the American Board of Family Medicine* 2010, **23**(5):598-605.

**80.** Nilsen P, Timpka T, Nordenfelt L, Lindqvist K: **Towards improved understanding of injury prevention program sustainability.** *Safety Science* 2005, **43**(10):815.

**81.** O'Loughlin J, Renaud L, Richard L, Gomez LS, Paradis G: **Correlates of the sustainability of community-based heart health promotion interventions.** *Prev Med* 1998, **27**(5):702-712.

**82.** Osganian SK, Hoelscher DM, Zive M, Mitchell PD, Snyder P, Webber LS: **Maintenance of effects of the Eat Smart School Food Service Program: results from the CATCH-ON study.** *Health Educ Behav* 2003, **30**(4):418-433.

**83.** Paine-Andrews A, Fisher JL, Campuzano MK, Fawcett SB, Berkley-Patton J: **Promoting sustainability of community health initiatives: an empirical case study.** *Health Promotion Practice* 2000, **1**:248-258.

**84.** Perlstein PH, Kotagal UR, Schoettker PJ, HD, Farrell MK, Gerhardt WE, Alfaro MP: **Sustaining the implementation of an evidence-based guideline for bronchiolitis.** *Arch Pediatr Adolesc Med* 2000, **154**(10):1001-1007.

**85.** Plochg T, Delnoij DM, Hoogedoorn NP, Klazinga NS: **Collaborating while competing? The sustainability of community-based integrated care initiatives through a health partnership.** *BMC Health Serv Res* 2006, **20**:6-37.

**86.** Pronovost PJ, Goeschel CA, Colantuoni E, Watson S, Lubomski LH, Berenholtz SM, Thompson DA, Sinopoli DJ, Cosgrove S, Sexton JB, Marsteller JA, Hyzy RC, Welsh R, Posa P, Schumacher K, Needham D: **Sustaining reductions in catheter related bloodstream infections in Michigan intensive care units: observational study.** *BMJ* 2010, **340**:c309-c313.

**87.** Rabin B, Nehl E, Elliott T, Deshpande A, Brownson R, Glanz K: **Individual and setting level predictors of the implementation of a skin cancer prevention program: a multilevel analysis.** *Implementation Science* 2010, **5**:40-53.

**88.** Rapoport A: **Sustainability of teachers’ international experiences: conditions for institutionalization of international program outcomes.** *Education Research International* , 2011:1–9.

**89.** Rau R, Rumpeltin C, Hoop R, Pfeiffer H, Drees J, Paas B, Schmitz-Buhl G, Geraedts M: **Five years "Healthy Lower Rhine…Against Stroke": implementation of a regional, intersectoral and sustainable public health program.** *Journal of Public Health* 2010, **18**(1):29-34.

**90.** Rog D, Boback N, Barton-Villagrana H, Marrone-Bennett P, Cardwell J, Hawdon J, Diaz J, Jenkins P, Kridler J, Reischl T: **Sustaining collaboratives: a cross-site analysis of The National Funding Collaborative on Violence Prevention.** *Eval Program Plann* 2004, **27**(3):249-261.

**91.** Rohrbach LA, Graham JW, Hansen WB: **Diffusion of a school-based substance abuse prevention program: predictors of program implementation.** *Prev Med* 1993, **22**(2):237-260.

**92.** Rollins A, Salyers M, Tsai J, Lydick J: **Staff turnover in statewide implementation of ACT: relationship with ACT fidelity and other team characteristics.** *Adm Policy Ment Health* 2009, **37**(5):417-426.

**93.** Rosenberg A, Hartwig K, Merson M: **Government-NGO collaboration and sustainability of orphans and vulnerable children projects in southern Africa.** *Eval Program Plann* 2008, **31**(1):51-60.

**94.** Rubin FH, Neal K, Fenlon K, Hassan S, Inouye SK: **Sustainability and scalability of the hospital elder life program at a community hospital.** *Journal of the American Geriatrics Society* 2011, **59**(2):359-365.

**95.** Ruch-Ross H, Keller D, Miller N, Bassewitz J, Melinkovich P: **Evaluation of community-based health projects: the healthy tomorrows experience.** *Pediatrics* 2008, **122**(3):e564-e572.

**96.** Sadof MD, Boschert KA, Brandt SJ, Motyl AP: **An analysis of predictors of sustainability efforts at the Inner-City Asthma Intervention sites: after the funding is gone.** *Annals of Allergy, Asthma & Immunology* 2006, **97**(S1):S31-S35.

**97.** Sanci L, Coffey C, Patton G, Bowes G: **Sustainability of change with quality general practitioner education in adolescent health: a 5-year follow-up.** *Med Educ* 2005, **39**:557-560.

**98.** Sanci LA, Coffey CM, Veit FC, Carr-Gregg M, Patton GC, Day N, Bowes G: **Evaluation of the effectiveness of an educational intervention for general practitioners in adolescent health care: randomised controlled trial.** *BMJ* 2000, **320**(7229):224-230.

**99.** Scheirer MA: **The life cycle of an innovation: adoption versus discontinuation of the fluoride mouth rinse program in schools.** *J Health Soc Behav* 1990, **31**(2):203-215.

**100.** Scheirer MA, Hartling G, Hagerman D: **Defining sustainability outcomes of health programs: Illustrations from an on-line survey.** *Eval Program Plann* 2008, **31**(4):335-346.

**101.** Schetzina KE, Dalton III WT, Pfortmiller DT, Robinson HF, Lowe EF, Stern HP: **The Winning With Wellness pilot project: rural Appalachian elementary student physical activity and eating behaviors and program implementation 4 years later.** *Fam Community Health* 2011*,* **34**(2):154-162.

**102.** Schoenwald SK, Carter RE, Chapman JE, Sheidow AJ: **Therapist adherence and organizational effects on change in youth behavior problems one year after multisystemic therapy.** *Adm Policy Ment Health* 2008, **35**(5):379-394.

**103.** Sebotsa MLD, Dannhauser A, Jooste PL, Joubert G: **Assessment of the sustainability of the iodine-deficiency disorders control program in Lesotho.** *Food And Nutrition Bulletin* 2007, **28**(3):337-347.

**104.** Simonsen B, Eber L, Black AC, Sugai G, Lewandowski H, Sims B, Myers D: **Illinois statewide positive behavioral interventions and supports: evolution and impact on student outcomes across years.** *Journal of Positive Behavior Interventions* 2012, **14**:5-16.

**105.** Stange KC, Goodwin MA, Zyzanski SJ, Dietrich AJ: **Sustainability of a practice-individualized preventive service delivery intervention.** *Am J Prev Med* 2003, **25**(4):296-300.

**106.** Steadman HJ, Cocozza JJ, Dennis DL, Lassiter MG, Randolph FL, Goldman H, Blasinsky M: **Successful program maintenance when federal demonstration dollars stop: the ACCESS program for homeless mentally ill persons.** *Adm Policy Ment Health* 2002, **29**(6):481-493.

**107.** Stetler CB, Ritchie JA, Rycroft-Malone J, Schultz AA, Charns MP: **Institutionalizing evidence-based practice: an organizational case study using a model of strategic change.** *Implementation Science* 2009, **4**:78.

**108.** Stevens B, Peikes D: **When the funding stops: do grantees of the Local Initiative Funding Partners Program sustain themselves?** *Eval Program Plann* 2006, **29**(2):153-161.

**109.** Stroul BA, Manteuffel BA: **The sustainability of systems of care for children’s mental health: lessons learned.** *Journal of Behavioral Health Services and Research* 2007, **34**(3):237-259.

**110.** Swain K, Whitley R, McHugo GJ, Drake RE: **The sustainability of evidence-based practices in routine mental health agencies.** *Community Ment Health J* 2009, **46**(2):119-129.

**111.** Thompson B, Lichtenstein E, Corbett K, Nettekoven L, Feng Z: **Durability of tobacco control efforts in the 22 Community Intervention Trial for Smoking Cessation (COMMIT) communities 2 years after the end of intervention.** *Health Educ Res* 2000, **15**(3):353-366.

**112.** Thorsen AV, Lassen AD, Tetens I, Hels O, Mikkelsen BE: **Long-term sustainability of a worksite canteen intervention of serving more fruit and vegetables.** *Public Health Nutrition* 2010*,* **13**(10):1647-1652.

**113.** Tibbits M, Bumbarger B, Kyler S, Perkins D: **Sustaining evidence-based interventions under real-world conditions: results from a large-scale diffusion project.** *Prev Sci* 2010, **11**(3):252-262.

**114.** Toledo Romani ME, Vanlerberghe V, Perez D, Lefevre P, Ceballos E, Bandera D, Baly Gil A, Van der Stuyft P: **Achieving sustainability of community-based dengue control in Santiago de Cuba.** *Soc Sci Med* 2007, **64**(4):976.

**115.** Ullrich S, McCutcheon H, Parker B: **Reclaiming time for nursing practice in nutritional care: outcomes of implementing Protected Mealtimes in a residential aged care setting.** *Journal of Clinical Nursing* 2010*,* **20**(9-10):1339-1348.

**116.** Visrutaratna S, Lindan CP, Sirhorachai A, Mandel JS: **" Superstar" and" model brothel": developing and evaluating a condom promotion program for sex establishments in Chiang Mai, Thailand.** *AIDS* 1995, **9**(1):S69-S75.

**117.** Wallin L, Bostrom A, Wikblad K, Ewald U: **Sustainability in changing clinical practice promotes evidence-based nursing care.** *J Adv Nurs* 2003, **41**(5):509–518.

**118.** Westrick SC, Breland ML: **Sustainability of pharmacy-based innovations: the case of in-house immunization services.** *Journal of the American Pharmacists Association* 2009, **49**(4):500-508.

**119.** Whitford DL, Roberts SH, Griffin S: **Sustainability and effectiveness of comprehensive diabetes care to a district population.** *Diabetic Medicine* 2004, **21**(11):1221-1228.

**120.** Woltmann EM, Whitley R, McHugo GJ, Brunette M, Torrey WC, Coots L, Lynde D, Drake RE: **The role of staff turnover in the implementation of evidence-based practices in mental health care.** *Psychiatr Serv* 2008, **59**(7):732-737.

**121.** Wong LC, Amega B, Barker R, Connors C, Dulla ME, Ninnal A, Cumaiyi MM, Kolumboort L, Currie BJ: **Factors supporting sustainability of a community-based scabies control program.** *Australasian Journal of Dermatology* 2002, **43**(4):274-277.

**122.** Wong ML, Chan KW, Koh D: **A sustainable behavioral intervention to increase condom use and reduce gonorrhea among sex workers in Singapore: 2-year follow-up.** *Prev Med* 1998, **27**:891–900.

**123.** Wright C, Catty J, Watt H, Burns T: **A systematic review of home treatment services: classification and sustainability.** *Soc Psychiatry Psychiatr Epidemiol* 2004, **39**(10):789-796.

**124.** Wright DB: **Care in the country: a historical case study of long-term sustainability in 4 rural health centers.** *Am J Public Health* 2009, **99**(9):1612-1618.

**125.** Xian Y, Pan W, Peterson ED, Heidenreich PA, Cannon CP, Hernandez AF, Friedman B, Holloway RG, Fonarow GC: **Are quality improvements associated with the Get With the Guidelines-Coronary Artery Disease (GWTG-CAD) program sustained over time?: A longitudinal comparison of GWTG-CAD hospitals versus non-GWTG-CAD hospitals.** *American Heart Journal* 2010, **159**(2):207-214.
